# Adipose tissue-specific ablation of PGC-1β impairs thermogenesis in brown fat

**DOI:** 10.1242/dmm.049223

**Published:** 2022-04-25

**Authors:** Jiří Funda, Josep A. Villena, Kristina Bardova, Katerina Adamcova, Illaria Irodenko, Pavel Flachs, Ivana Jedlickova, Eliska Haasova, Martin Rossmeisl, Jan Kopecky, Petra Janovska

**Affiliations:** 1 Laboratory of Adipose Tissue Biology, Institute of Physiology of the Czech Academy of Sciences, 142 20 Prague, Czech Republic; 2 Department of Physiology, Faculty of Science, Charles University in Prague, 128 44 Prague, Czech Republic; 3 Laboratory of Metabolism and Obesity, Vall d'Hebron-Institut de Recerca, Universitat Autònoma de Barcelona, 08035 Barcelona, Spain; 4 Centro de Investigación Biomédica en Red de Diabetes y Enfermedades Metabólicas Asociadas, Instituto de Salud Carlos III, 28029 Madrid, Spain; 5 Research Unit for Rare Diseases, Department of Paediatrics and Inherited Metabolic Disorders, First Faculty of Medicine, Charles University and General University Hospital, 128 08 Prague, Czech Republic

**Keywords:** Lipid metabolism, OPA1, Mice, Adrenergic control

## Abstract

Impaired thermogenesis observed in mice with whole-body ablation of peroxisome proliferator-activated receptor-γ coactivator-1β (PGC-1β; officially known as PPARGC1B) may result from impaired brown fat (brown adipose tissue; BAT) function, but other mechanism(s) could be involved. Here, using adipose-specific PGC-1β knockout mice (PGC-1β-AT-KO mice) we aimed to learn whether specific PGC-1β ablation in adipocytes is sufficient to drive cold sensitivity. Indeed, we found that warm-adapted (30°C) mutant mice were relatively sensitive to acute cold exposure (6°C). When these mice were subjected to cold exposure for 7 days (7-day-CE), adrenergic stimulation of their metabolism was impaired, despite similar levels of thermogenic uncoupling protein 1 in BAT in PGC-1β-AT-KO and wild-type mice. Gene expression in BAT of mutant mice suggested a compensatory increase in lipid metabolism to counteract the thermogenic defect. Interestingly, a reduced number of contacts between mitochondria and lipid droplets associated with low levels of L-form of optic atrophy 1 was found in BAT of PGC-1β-AT-KO mice. These genotypic differences were observed in warm-adapted mutant mice, but they were partially masked by 7-day-CE. Collectively, our results suggest a role for PGC-1β in controlling BAT lipid metabolism and thermogenesis.

This article has an associated First Person interview with the first author of the paper.

## Abstract (precis)

**Summary:** Adipose tissue-specific ablation of PGC-1β impairs thermogenesis in murine brown fat owing to a reduced number of contacts between mitochondria and lipid droplets, possibly involving the L-form of optic atrophy 1 protein.

## INTRODUCTION

The main function of brown adipose tissue (BAT) is the maintenance of body temperature via non-shivering thermogenesis (NST), which is mediated by uncoupling protein 1 (UCP1) (Golozoubova et al., 2006). BAT is present only in mammals. In rodents, interscapular BAT quantitatively represents the most important UCP1-containing fat depot and, therefore, plays a main role in UCP1-mediated thermogenesis (reviewed by Cannon and Nedergaard, 2004). UCP1 is also present in inducible adipocytes interspersed in white adipose tissue (WAT) depots, called brite/beige adipocytes (Petrovic et al., 2010; Wu et al., 2012). BAT is vital for homeothermy in human neonates, which is compromised in prematurely born babies. Also, in accordance with the resistance to obesity of mice harboring transgenic overexpression of UCP1 in WAT (Kopecky et al., 1995) and the involvement of UCP1 in diet-induced thermogenesis (von Essen et al., 2017), it is often speculated that NST mediated by UCP1 could serve as a target for obesity treatment. BAT ‘whitening’ and decrease of its activity have been described in obesity, during adaptation to thermoneutral conditions, or due to leptin receptor deficiency, β-adrenergic signaling impairment or deficiency of adipose triglyceride lipase (ATGL; officially known as PNPLA2) (Kotzbeck et al., 2018). Energy demands for NST strongly depend on ambient temperature and, therefore, they are negligible at thermoneutral conditions (Cui et al., 2016). Under these environmental conditions, the thermogenic program in BAT is downregulated (Cui et al., 2016). Still, in mice maintained at thermoneutrality, despite a lower food intake, body weight was unchanged although fat mass tended to be increased. However, control of BAT activity under thermoneutral conditions requires better characterization.

In the activation and regulation of NST, transcription factors and members of the peroxisome proliferator-activated receptor-γ coactivator-1 family (PGC-1α and PGC-1β; officially known as PPARGC1A and PPARGC1B, respectively) are involved. During cold exposure (CE), adrenergic stimulation strongly upregulates *Ppargc1a* expression to activate the *Ucp1* gene through co-activation of peroxisome proliferator-activated receptor alpha (product of the gene *Ppara*). Supporting the crucial role of PGC-1α in regulating NST, mice lacking PGC-1α are cold sensitive (reviewed by Villena, 2015), in part as a result of the failure to increase *Ucp1* expression in response to low environmental temperatures. Although *Ppargc1b* expression, in contrast to *Ppargc1a*, is not increased during CE (Lin et al., 2002), mice with whole-body *Ppargc1b* ablation exhibit dysregulated and compromised expression of mitochondrial genes in BAT, especially when exposed to cold conditions, despite a compensatory increase in *Ppargc1a* expression (Lelliott et al., 2006; Sonoda et al., 2007). Still, the role of PGC-1β in maintaining body temperature remained unclear, with different studies providing conflicting results. In the study by Lelliott et al. (2006), whole-body ablation of PGC-1β had no effect on survival of the mutant mice in cold conditions, even though there was a reduction in mitochondrial gene expression in BAT and impairment of norepinephrine (NE)-stimulated energy expenditure. By contrast, in a study by Sonoda et al. (2007), PGC-1β-ablated mice also showed reduced mitochondrial gene expression in BAT but, in addition, low resistance to acute CE.

PGC-1α and PGC-1β are abundant in tissues with high mitochondrial mass, such as heart, skeletal muscle and BAT. The high sequence similarity between PGC-1α and PGC-1β may explain partial redundancy/replaceability in their functions, but their responses to physiological stimuli differ. Whereas in skeletal muscle the function of both co-activators appears to be redundant, in the liver *Ppargc1a* mRNA increases during fasting, in contrast to *Ppargc1b* expression, which is induced in response to dietary saturated fatty acids (reviewed by Villena, 2015). Thus, PGC-1α is involved in the regulation of gluconeogenesis in the liver, unlike PGC-1β, which is a key regulator of hepatic lipogenesis. As noted above, mice with whole-body deletion of PGC-1β show a variable phenotypic responsiveness in terms of cold tolerance. This could reflect impaired BAT thermogenic function or a broad effect of PGC-1β deletion on the metabolism of several tissues that contribute to the maintenance of body temperature.

The aim of our current study was to determine unambiguously the role of PGC-1β in adipose tissue under conditions that are associated with different demands on thermogenesis, i.e. in a thermoneutral environment or during CE. To this end, a new mouse model with *Ppargc1b* deletion specifically in adipocytes (PGC-1β-AT-KO) was generated and characterized. Our results demonstrate an important role of PGC-1β in controlling BAT lipid metabolism and thermogenesis, reflecting the transcriptional control by PGC-1β of the expression of the protein machinery that mediates both (1) adrenergic control of lipolysis and (2) contacts between mitochondria and lipid droplets.

## RESULTS

**Fig. 1.**
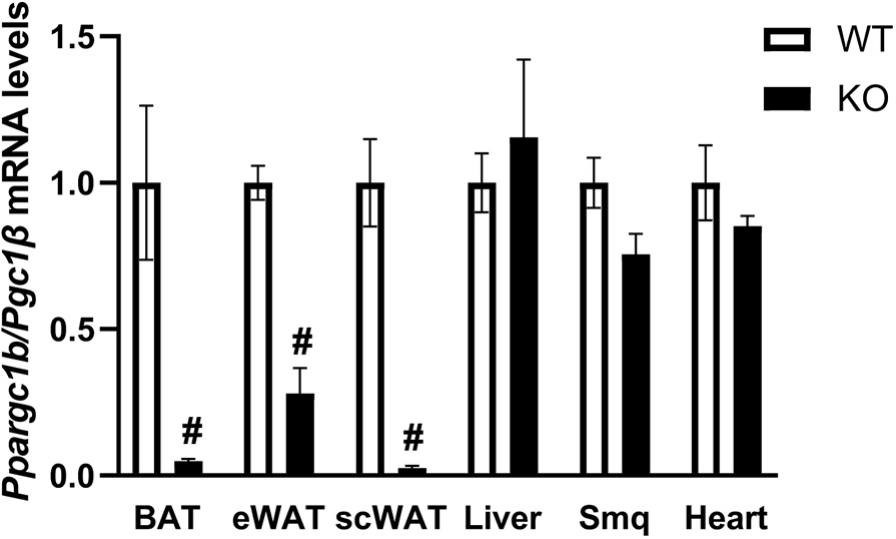
**Confirmation by qPCR of adipose tissue-specific deletion of the *Ppargc1b* gene.** Tissues collected from mice subjected to 7-day-CE were used. BAT, interscapular brown adipose tissue; eWAT, epididymal white adipose tissue; scWAT, subcutaneous-inguinal white adipose tissue; KO, PGC-1β-AT-KO mice; Smq, skeletal muscle quadriceps; WT, wild-type mice. (BAT, *n*=8; other tissues, *n*=5). Data are mean±s.e.m. ^#^*P*<0.05 (significant difference between genotype; *t*-test, unpaired, two-tailed).

Mutant PGC-1β-AT-KO mice were generated on a C57BL/J background using the adiponectin gene promoter to secure fat-specific deletion of *Ppargc1b* (see Materials and Methods). The specificity of the deletion was confirmed at the gene expression level. As expected, almost complete abolishment of *Ppargc1b* mRNA expression was observed in interscapular BAT (Figs 1 and 3A), as well as in subcutaneous inguinal white adipose tissue (scWAT), whereas a ∼70% decrease in *Ppargc1b* transcript levels was observed in epididymal WAT (eWAT). In extra-adipose tissues of PGC1-β-AT-KO mice, such as skeletal muscle, heart and liver, *Ppargc1b* transcript levels were not affected (Fig. 1), confirming the presence of the deletion only in adipose tissue. Mutant and wild-type (WT) mice showed similar responses to both high-fat feeding and calorie restriction regarding body weight, adiposity and plasma markers of lipid metabolism (Table S1).

Both homozygous PGC-1β-AT-KO and WT mice maintained at thermoneutral temperature of 30°C were subjected to CE (6°C) for 2 or 7 days. At 30°C, mutant mice did not differ from their WT littermates in body weight, food consumption, weight of WAT depots, liver and quadriceps muscle weight, or plasma levels of triacylglycerols (TAG), non-esterified fatty acids (NEFA) and insulin (Table 1). However, BAT weight was higher (∼1.6-fold) in PGC-1β-AT-KO than in WT mice, owing to the higher total content of TAG in BAT of mutant mice, whereas total tissue protein was similar in mice of both genotypes. During CE, the differences in BAT mass decreased, and the total amount of TAG in the tissue decreased to reach similar levels in mice of both genotypes whereas total tissue protein content doubled independently of the genotype. Mice of both genotypes showed increased food consumption and decreased body weight gain, with more pronounced changes observed in PGC-1β-AT-KO mice after only 2 days of CE. As previously observed in mice (Flachs et al., 2017), plasma levels of both TAG and NEFA declined transiently after 2 days and returned to the original values after 7 days of CE, although only partial recovery occurred in PGC-1β-AT-KO mice. As a result, both TAG and NEFA levels after 7-days of CE were lower in PGC-1β-AT-KO than in WT mice (Table 1).

**Table 1.**
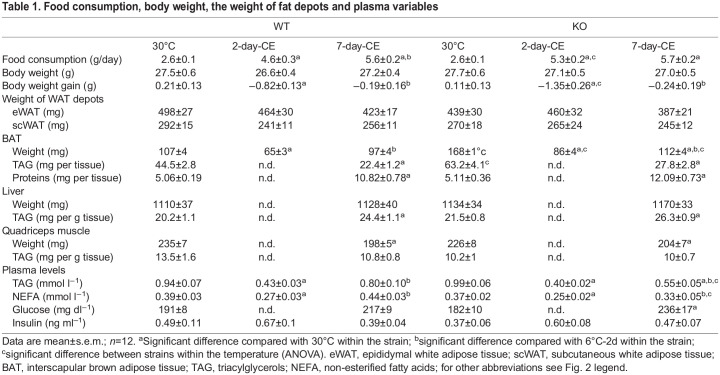
Food consumption, body weight, the weight of fat depots and plasma variables

**Fig. 2.**
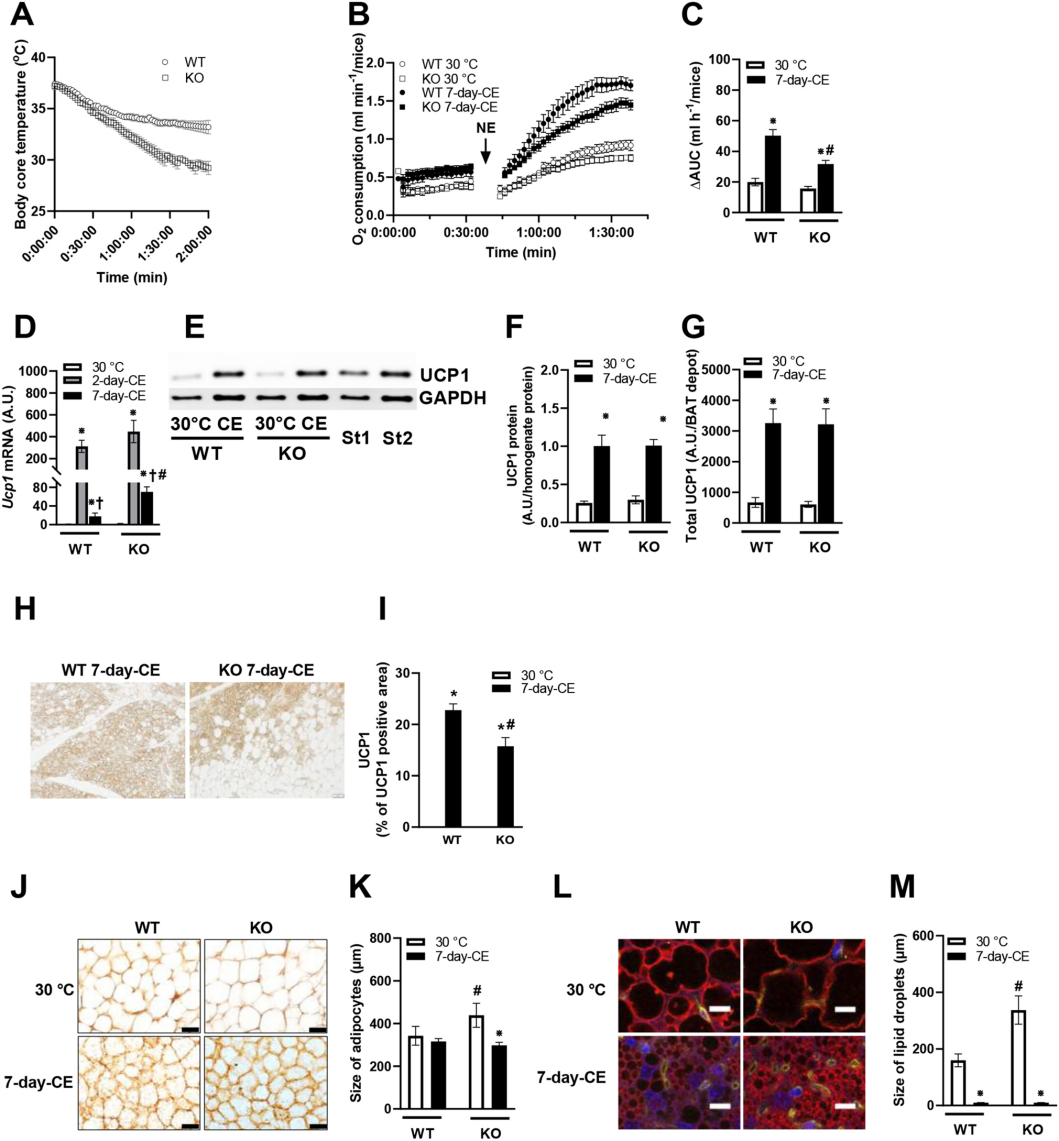
**Effect of PGC-1β ablation on NST, UCP1 expression in interscapular BAT and scWAT, and adipose tissue histology.** (A) Cold-tolerance test in animals exposed to 6°C showing development of hypothermia in PGC-1β-AT-KO mice adapted to 30°C; *n*=7. (B,C) O_2_ consumption during NE test suggesting impaired UCP1-mediated thermogenesis in PGC-1β-AT-KO mice, especially after 7-day-CE; NE-stimulated oxygen consumption was measured for 90 min after NE injection and basal consumption subtracted (B; see also Materials and Methods, ‘Indirect calorimetry’ section). WT 30°C, *n*=6; other groups *n*=9. (D) Effect of CE on *Ucp1* expression in BAT (A.U., arbitrary units); *n*=14. (E) Representative blots of UCP1 and glyceraldehyde-3-phosphate dehydrogenase (GAPDH; used as a loading control). Standards St1 and St2 (St1 is a half concentration of St2) were used for comparison of signals on different blots. (F) Amount of UCP1 per 2 µg of BAT homogenate protein. (G) UCP1 protein levels recalculated to represent the whole interscapular BAT depot (A.U.) in mice adapted to thermoneutral conditions or subjected to 7-day-CE; warm-adapted groups *n=*8; CE groups *n*=12. (H,I) UCP1 detection (H) and the percentage of UCP1-positive area in dorsolumbal scWAT (I) by immunohistochemical analysis. (J-M) Size of adipocytes (J,K) and lipid droplets (L,M) in BAT of mice adapted to thermoneutral conditions or subjected to 7-day-CE, based on immunohistological analysis; *n=*5. Scale bars: 100 µm (J); 20 μm (L). The size of lipid droplets was assessed using anti-perilipin antibody (red), DAPI was used for visualization of cell nuclei (violet, not quantified) and isolectin for capillaries (yellow, not quantified). Data are mean±s.e.m. Statistical analysis was performed using *t*-test (A) or two-way (B-M) ANOVA (*P*<0.05). *Significant difference compared to 30°C within the genotype; ^†^significant difference compared to 2 days at 6°C, within the genotype; ^#^significant difference between genotypes within the same temperature. KO, PGC-1β-AT-KO mice; WT, wild-type mice; 30°C, mice adapted to thermoneutral conditions; 2-day-CE, mice exposed to cold conditions for 2 days; 7-day-CE, mice exposed to cold conditions for 7 days. ΔAUC, delta of area under the curve for oxygen consumption.

Next, we focused on the thermogenic capacity of mice. When animals adapted to thermoneutral conditions were acutely exposed to 6°C, their core body temperature declined, with more pronounced hypothermia observed in PGC-1β-AT-KO mice between the first and second hour in the cold compared with WT controls. After 2 h in the cold, the core body temperature of most of PGC-1β-AT-KO mice approached 28°C (Fig. 2A), whereas that of WT mice stabilized around 33°C, indicating impaired thermogenesis in PGC1β-AT-KO mice. However, despite a decrease in body temperature during the first 2 h in the cold (Fig. 2A), these animals were able to survive the cold stress.

Stimulation of NST in response to acute CE, as well as the increase in NST capacity elicited by cold adaptation, is largely mediated by the adrenergic system (Cannon and Nedergaard, 2004). It is estimated that 60-80% of adrenergic stimulation of thermogenesis in mice depends on UCP1 (Granneman et al., 2003). Hence, to evaluate the role of NST in BAT in the effect of PGC-1β loss on thermal homeostasis of the mice, NE-stimulated oxygen consumption, corresponding to energy expenditure, was measured in anesthetized mice at 33°C (Golozoubova et al., 2006), for 30 min before and 60 min after NE injection (Fig. 2B). Animals adapted to thermoneutral conditions of 30°C as well as mice exposed to 6°C for 7 days were used. Under basal (un-stimulated) conditions, mice adapted to the cold tended to consume more oxygen compared with mice adapted to thermoneutral conditions, but with no significant differences between mutant and WT mice. As expected, administration of NE stimulated oxygen consumption, which was greater in mice subjected to 7-day-CE. Importantly, the response to NE stimulation was significantly reduced in PGC-1β-AT-KO compared with WT mice, with only a trend toward a reduced response observed in mutant mice maintained in thermoneutrality (Fig. 2C). These results suggested that NST in BAT rather than another thermogenic mechanism, i.e. shivering thermogenesis (Blondin and Haman, 2018), was affected by the fat-specific ablation of PGC-1β.

To uncover the molecular mechanism underlying impaired thermogenesis in PGC-1β-AT-KO mice, the possible involvement of UCP1 was first characterized. In interscapular BAT, expression of *Ucp1* mRNA was negligible in mice adapted to thermoneutral conditions and was dramatically induced by CE. This effect was more pronounced after 2 days compared with 7 days of CE. However, the expression was higher in PGC-1β-AT-KO compared with WT mice subjected to 7-day-CE (Fig. 2D), suggesting a compensatory response to the defect in thermogenesis at the gene expression level. In contrast, UCP1 protein levels measured by western blot in BAT homogenates (Fig. 2E) similarly increased in response to CE, independently of genotype (Fig. 2E,F). Furthermore, the total amount of UCP1 in BAT, recalculated using the BAT protein data (Table 1), was similar in mice of both genotypes and was about 2-fold higher in mice exposed to cold conditions for 7 days, compared with the warm-adapted animals (Fig. 2G). Next, we focused on UCP1 contained in brite/beige adipocytes, which are typically found in scWAT (Petrovic et al., 2010; Wu et al.). In scWAT homogenates prepared from warm-adapted WT mice, negligible amounts of UCP1 could be detected; the UCP1 content was increased in response to 7-day-CE, but even under these conditions UCP1 content recalculated per whole fat depot was two orders of magnitude lower in scWAT compared with interscapular BAT (data not shown). Immunohistochemical analysis of scWAT detected no UCP1 in warm-adapted mice, regardless of the genotype (not shown). However, UCP1-positive adipocytes were found in mice subjected to 7-day-CE (Fig. 2H). Their content, evaluated as the area of UCP1-positive cells, was about 1.6-fold lower in mutant compared with WT mice (Fig. 2I). The above data suggested that insufficient capacity for adaptive NST in BAT of PGC-1β-AT-KO mice, demonstrated by whole-body oxygen consumption measurements (see above), is not due to the inability of mutant mice to induce UCP1 in response to cold conditions.

The relative enlargement of BAT in PGC-1β-AT-KO mice, observed primarily in animals adapted to thermoneutral conditions (see above, Table 1), reflected rather the TAG than the protein content of the tissue (Table 1). This prompted us to characterize immunohistologically the size of BAT adipocytes and their intracellular lipid droplets. In warm-adapted mice, brown adipocytes adopted a white-like morphology with one or very few large lipid droplets. In accordance with the TAG accumulation (see above), adipocytes (Fig. 2J,K) and their lipid droplets (Fig. 2L,M) were larger in PGC-1β-AT-KO mice than in WT controls. After 7-day-CE, adipocytes in PGC-1β-AT-KO mice, but not in WT animals, became smaller, and lipid droplet size was extremely reduced, to the same level in both genotypes. These results suggest an impairment of brown adipocyte metabolism in PGC-1β-AT-KO mice that promoted lipid accumulation within these cells. This feature was apparent exclusively under thermoneutral conditions, but not in mice subjected to CE.

**Fig. 3.**
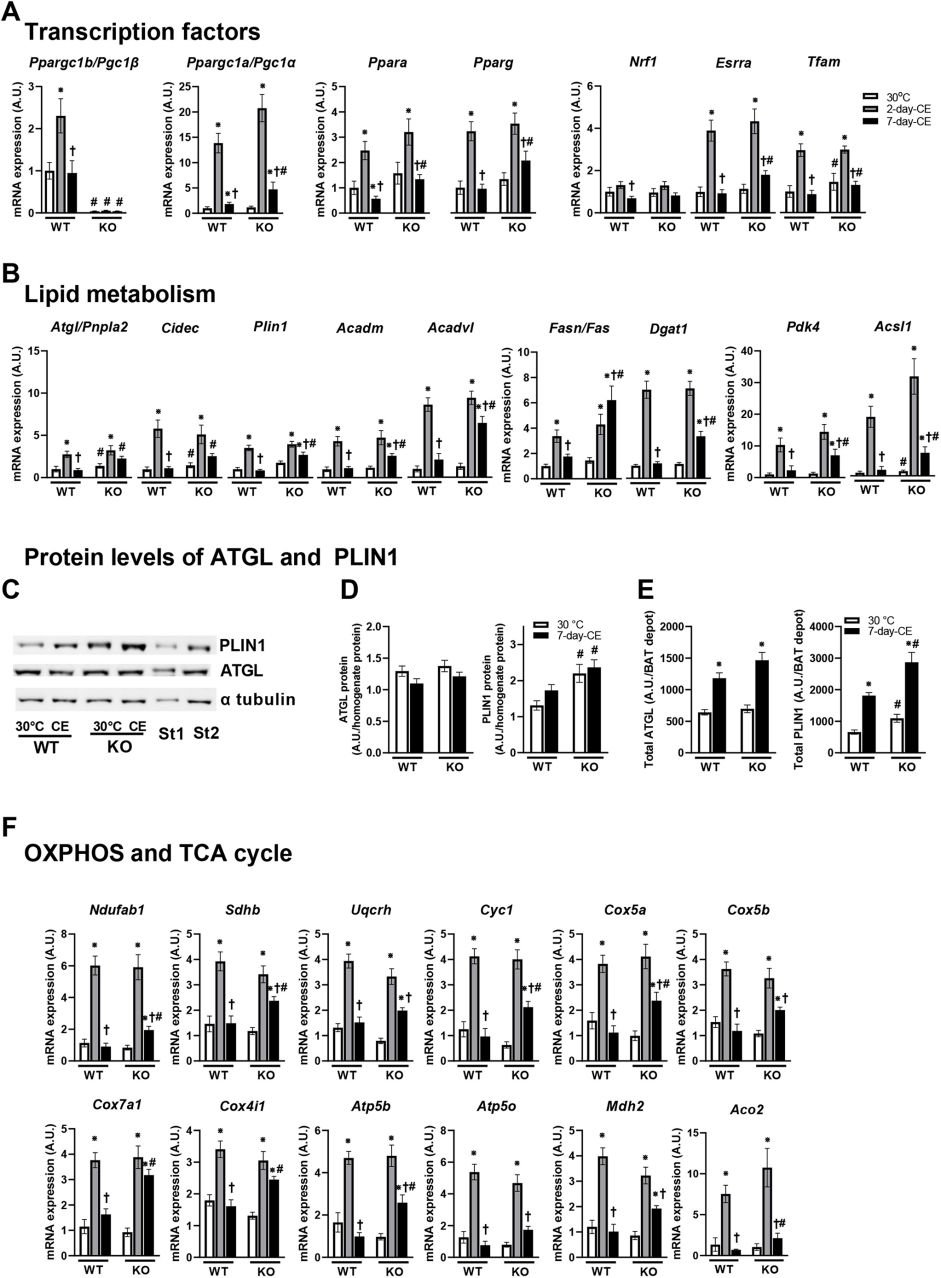
**Genes and proteins connected to lipid and mitochondrial metabolism.** (A-F) Effect of PGC-1β ablation on the expression of selected genes (A,B,F) and proteins (C-E) in BAT. C shows representative blots with α-tubulin as loading control. *n=*8. Data are mean±s.e.m. Statistical analysis was performed using two-way ANOVA (*P*<0.05). *Significant difference compared to 30°C within the genotype; ^†^significant difference compared to 2 days at 6°C, within the genotype; ^#^significant differences between genotype within the same temperature. KO, PGC-1β-AT-KO mice; WT, wild-type mice; 30°C, mice adapted to thermoneutral conditions; 2-day-CE, mice exposed to cold conditions for 2 days; 7-day-CE, mice exposed to cold conditions for 7 days.

To characterize in detail the effect of PGC-1β ablation on BAT metabolism, the mRNA levels of selected gene markers of lipid and mitochondrial metabolism and their transcriptional regulators were quantified in interscapular BAT (Fig. 3). Although 2-day-CE activated expression of almost all genes analyzed, in WT mice the levels of most of these normalized after 7 days of CE. Thus, transient upregulation of gene expression in response to 2-day-CE was found in both genotypes. However, in the case of 7-day-CE, ablation of PGC-1β was associated with relatively high expression levels. This was also true for the genes for transcription factors with more general roles in lipid metabolism and mitochondrial functions, such as *Ppargc1a*, *Ppara* and *Pparg*, as well as factors involved more specifically in mitochondrial biogenesis, such as estrogen related receptor alpha (*Esrra*) and transcription factor A (*Tfam*; Fig. 3A).

The expression pattern of the transcription factors genes then corresponded to the expression of their target genes, i.e. markers of lipid metabolism, including: (1) *Atgl* (*Pnpla2*), encoding the key lipolytic enzyme in adipocytes, (2) *Cidec*, encoding cell death-inducing DNA fragmentation factor, which promotes lipid droplet formation in adipocytes, (3) *Plin1*, encoding perilipin 1, which restricts of lipolysis mediated by ATGL, under basal conditions but activates under adrenergically stimulated conditions, (4) *Acadm* and *Acadvl1*, encoding acyl-CoA medium chain and very long chain dehydrogenase, respectively, which catalyze the first step in fatty acid oxidation, (5) *Fasn* (also known as *Fas*), encoding fatty acid synthase, which is essential for *de novo* lipogenesis, and (6) *Dgat1*, encoding diacylglycerol O-acyltransferase 1, involved in fatty acid re-esterification (Fig. 3B). Moreover, a similar pattern of gene expression was observed with pyruvate dehydrogenase kinase 4 (*Pdk4*), which inhibits pyruvate dehydrogenase and thus promotes fatty acid oxidation, and Acyl-CoA synthetase long-chain family member 1 (*Acsl1*), involved in lipid biosynthesis and fatty acid degradation (Fig. 3B). These results indicate a general activation of lipid metabolism in mice with PGC-1β ablation subjected to 7-day-CE compared with WT controls. Upregulation of *Atgl*, *Cidec* and *Acsl1* was observed in PGC-1β-AT-KO mice even in a thermoneutral environment (Fig. 3B), consistent with the relatively high mass of BAT in PGC-1β-AT-KO mice under these conditions (see Table 1 and Discussion). In contrast with the gene expression data, quantification of proteins in BAT homogenates using western blotting showed similar levels ATGL in mice of both genotypes, independent of CE, and quantification of PLIN1 confirmed the gene expression data (Fig. 3C,D). When recalculated per whole interscapular BAT, the content of these two proteins was increased in response to 7-day-CE compared with the warm-adapted mice. Moreover, PLIN1 content was higher in mutant than in WT mice independent of CE (Fig. 3E).

Furthermore, all the genes encoding proteins involved in oxidative phosphorylation (OXPHOS) and the tricarboxylic acid (TCA) cycle were similarly transiently upregulated after 2-day-CE in WT and PGC-1β-AT-KO mice. Expression of most of them normalized after 7-day-CE, but in general their mRNA levels remained higher in PGC-1β-AT-KO mice (Fig. 3F), consistent with compensatory upregulation of *Ppargc1a*, *Esrra* and *Tfam* in these mice (Fig. 3A). However, no major effect of PGC-1β ablation on OXPHOS proteins content in either BAT homogenates or isolated BAT mitochondria from mice subjected to 7-day-CE could be detected using western blots (Fig. S1).

**Fig. 4.**
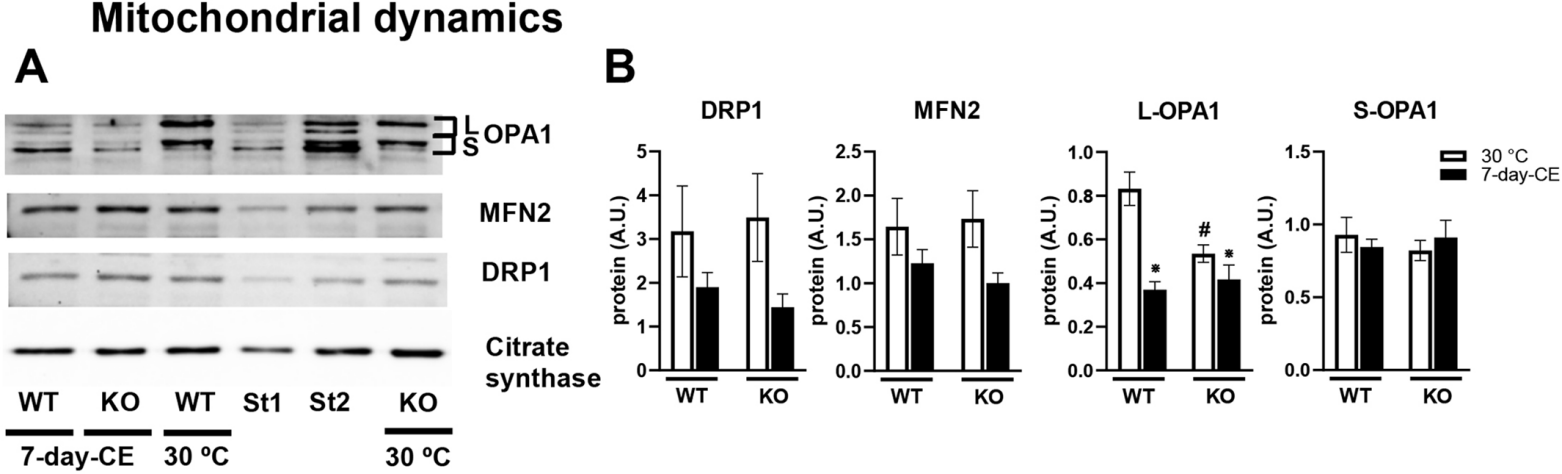
**Effect of PGC-1β ablation on the proteins involved in mitochondrial dynamics.** (A) Representative western blots detecting OPA1, MFN2, DRP1 and citrate synthase (used as a loading control) in BAT homogenates. Data are expressed in arbitrary units (A.U.; per 2 µg of total protein). St1 (half the concentration of St2) and St2 were used for comparison of signals between different blots. (B) Proteins involved in mitochondrial dynamics. *n*=8; for L- and S-forms of OPA1, *n*=6. The sum of two upper signals were used for quantification of L-OPA1 and the sum of three lower signals were used for quantification of S-OPA1 (see Fig. 4A). Data are mean±s.e.m. Statistical analysis was performed using two-way ANOVA (*P*<0.05). *Significant differences in comparison to 30°C within the genotype; ^#^significant differences between genotypes within temperature. KO, PGC-1β-AT-KO mice; WT, wild-type mice; 7-day-CE, mice exposed to cold conditions for 7 days; 30°C, mice adapted to thermoneutral conditions.

We further focused on proteins controlling mitochondrial dynamics, which are important for mitochondrial function and cellular energy metabolism, and possibly also for adrenergic regulation of lipolysis (reviewed by Mishra and Chan, 2016), namely (1) dynamin-related protein 1 (DRP1; officially known as DNM1L), a GTPase regulating mitochondrial fission (Mishra and Chan, 2016), (2) mitofusin 1 and 2 (MFN1, MFN2), proteins involved in fusion of outer mitochondrial membranes, and (3) optic atrophy 1 protein (OPA1), which regulates fusion of inner mitochondrial membranes, mitochondrial functions (Lee et al., 2017) and lipolysis (Pidoux et al., 2011) (see Discussion). In interscapular BAT of mice of both genotypes, expression of the genes for all these proteins was transiently upregulated during CE, reaching the highest levels in mice subjected to 2-day-CE, and it mostly normalized between 2 and 7 days of CE (Fig. S2). These proteins were quantified using western blots in BAT homogenates prepared from warm-acclimated mice and animals subjected to 7-day-CE (Fig. 4A). Levels of DRP1 and MFN2 tended to be lower in the CE compared with the warm-adapted mice, with no differences between genotypes (Fig. 4B). In the case of OPA1, both forms of the protein were detected, i.e. long form (L-OPA1) and the proteolytically cleaved short form (S-OPA1; Fig. 4), with both forms present in several isoforms (Lee et al., 2017; Quiros et al., 2012). In WT mice, L-OPA1 levels decreased in response to 7-day-CE by about 1.4-fold (Fig. 4B), consistent with OPA1 cleavage and mitochondrial fragmentation after adrenergic stimulation of lipolysis (Wikstrom et al., 2014). Although ablation of PGC-1β was associated with a decrease in L-OPA1 under thermoneutral conditions, L-OPA1 levels did not differ between mutant and WT mice after 7-day-CE (Fig. 4). Thus, in contrast to WT mice, L-OPA1 levels in PGC-1β-AT-KO mice did not respond to CE. In contrast, S-OPA1 levels were not affected either by temperature or by genotype (Fig. 4). These results are in agreement with the irreplaceable function of PGC-1β under basal (thermoneutral) conditions and suggest that impaired control of BAT lipid metabolism in PGC-1β-KO mice may reflect compromised L-OPA1 function.

**Fig. 5.**
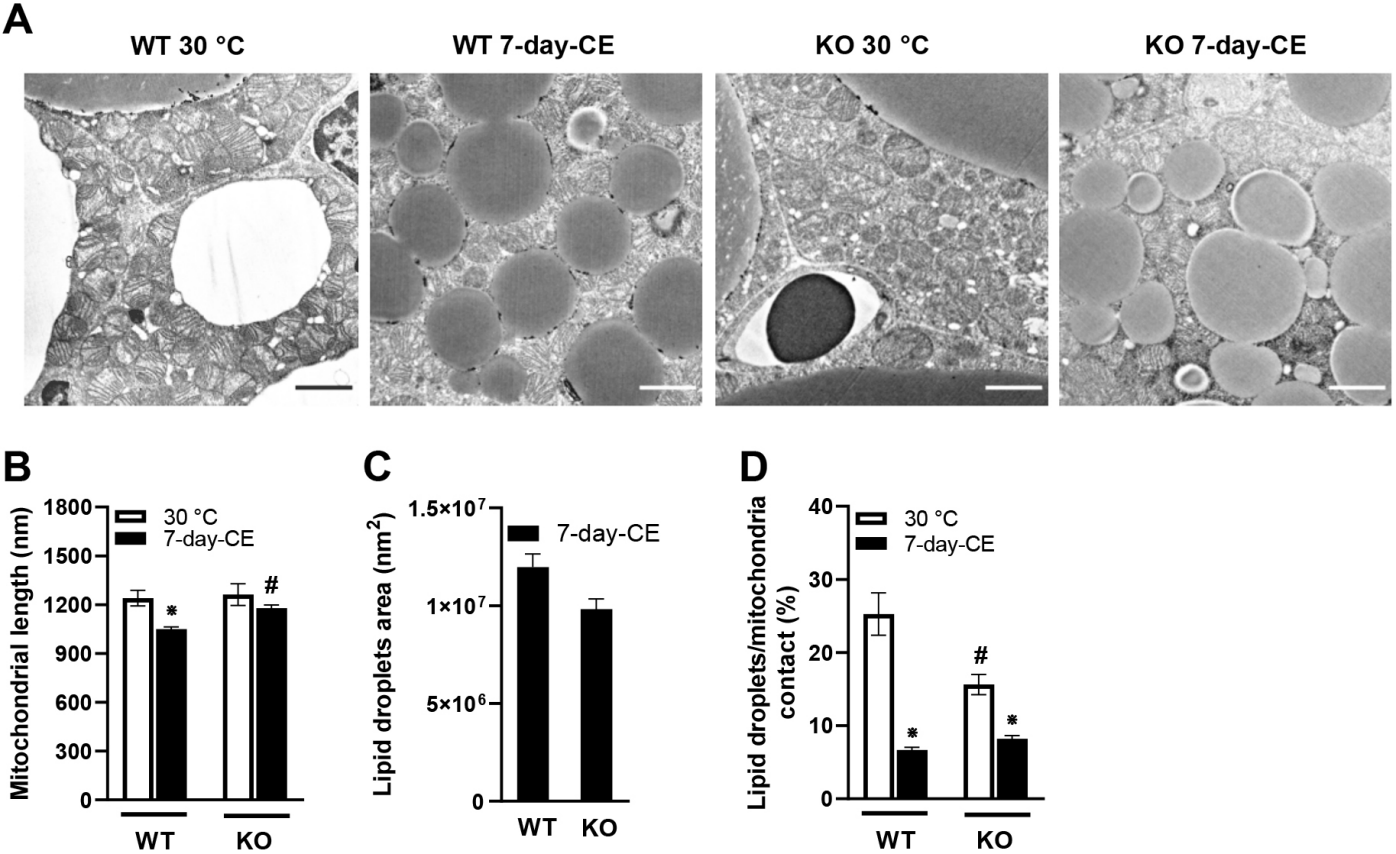
**PGC-1β ablation reduced contacts between mitochondria and lipid droplets in interscapular BAT.** (A) Representative electron microscopy images of BAT. Scale bars: 2 µm. (B-D) Quantification of the length of mitochondria (B), size of lipid droplets (C) and the connection between mitochondria and lipid droplets (D). Mice were adapted to thermoneutral conditions (A,B,D) or subjected to 7-day-CE, (A-D); *n=*5. Data are mean±s.e.m. Statistical analysis was performed using two-way ANOVA (*P*≤0.05). *Significant difference compared to 30°C within the genotype; ^#^significant difference between the genotype within temperature. KO, PGC-1β-AT-KO mice; WT, wild-type mice; 30°C, mice adapted to thermoneutral conditions, 7-day-CE, mice exposed to cold conditions for 7 days.

Electron microscopy analysis of interscapular BAT (Fig. 5A) showed a reduction in mitochondrial length in adipocytes from WT mice in response to 7-day-CE (Fig. 5B), consistent with the L-OPA1 data (Fig. 4B), and only a trend toward decreased mitochondrial length in PGC-1β-KO mice (Fig. 5B). Lipid droplet area was reduced in response to CE in both genotypes (area of lipid droplets in warm-adapted mice was not measured, see Materials and Methods), with a tendency toward smaller droplets in PGC-1β-AT-KO mice (Fig. 5C). Moreover, quantitative analysis of the area of contact between mitochondria and lipid droplets in BAT adipocytes from WT mice revealed a ∼3.8-fold reduction in response to 7-day-CE (Fig. 5D). Compared with WT animals, PGC-1β ablated mice showed ∼40% reduction in contact area already under thermoneutral conditions, and a much smaller relative reduction in response to 7-day-CE, resulting in similar contact area in mice of both genotypes in the CE mice (Fig. 5D). Thus, the pattern of changes in the area of contacts between mitochondria and lipid droplets in BAT adipocytes was similar to that of L-OPA1 levels (Fig. 4B). These findings support the notion that impairment of mitochondria-lipid droplet contacts in brown adipocytes is related to compromised function of OPA1 in PGC-1β-AT-KO mice observed under thermoneutral conditions. This could lead to the observed impairment in utilization of lipids contained in intracellular lipid droplets for UCP1-mediated thermogenesis.

## DISCUSSION

Our results provide evidence for a specific role of PGC-1β in adipocytes in controlling lipid metabolism in BAT, both under basal conditions in a thermoneutral environment and during thermoregulatory thermogenesis. They demonstrate that this function of PGC-1β reflects its interaction with the protein machinery involved in both the control of lipolysis and contacts between mitochondria and lipid droplets. This specific role of PGC-1β cannot be completely replaced by PGC-1α, despite the adaptive upregulation of gene expression of the latter transcription factor in PGC-1β-AT-KO.

Our experiments using mice with specific deletion of PGC-1β in adipose tissue support the previously observed hypothermia and BAT malfunction in mice with whole-body PGC-1β ablation (Sonoda et al., 2007). That NE-stimulated energy expenditure was affected here provides evidence for impairment of adrenergic NST. Moreover, the altered interaction between mitochondria and lipid droplets points to NST occurring in BAT (see below), despite the normal UCP1 protein content in BAT and its adaptive increase following CE (Fig. 2E-G). However, BAT-specific deletion of PGC-1β would be required to understand fully the underlying mechanism. Although the defective thermogenesis is evident during acute exposure to low temperatures, the animals manage to survive and can adapt to long-term cold exposure, suggesting that the impairment of BAT thermogenesis due to PGC-1β ablation is compensated for by other thermogenic mechanism(s) not related to BAT, similar to observations in mice with complete UCP1 ablation (Liu et al. 2003). If this were not the case, the mutant mice would be unlikely to survive several days in the cold. Either shivering (Blondin and Haman, 2018) or UCP1-indendent NST (Flachs et al., 2013; Solinas et al., 2004; Ukropec et al., 2006), or both, could be involved.

The strong reduction in expression of BAT genes related to mitochondrial energy metabolism observed in mice with whole-body deletion of PGC-1β (Lelliott et al., 2006; Sonoda et al., 2007) and in mice with PGC-1β deletion using the *Fabp4* (also known as AP2) promoter (ap2-PGC1β-KO; deletion specific for adipose tissue and macrophages) maintained at 30°C (Enguix et al., 2013) was not observed here using PGC-1β-AT-KO mice. Instead, the mutant mice in our study showed only a trend towards reduced mitochondrial gene expression at thermoneutrality (Fig. 3F). The compensatory increase in *Ppargc1a* expression in our PGC-1β-AT-KO mice maintained at standard ambient temperature of 20°C (Fig. S3) or in animals subjected to CE (Fig. 3A) could explain the minimal effect of PGC-1β loss on mitochondrial gene expression (Fig. 3F). This is in contrast with the previous findings involving ap2-PGC1β-KO mice maintained at 21°C (Enguix et al., 2013). Under these conditions, no compensatory increase in *Ppargc1a* was observed, and therefore mitochondrial gene expression was severely decreased. These differences could reflect the conditions under which the mice were maintained prior to the experiments, and the length of adaptation to warmth and cold, respectively. Thus, animals in our study were born and maintained at 20°C until at least 6 weeks of age (see ‘Animal design’ under Materials and Methods, and Flachs et al., 2017), i.e. under conditions of mild CE (Cannon and Nedergaard, 2011), and thereafter adapted to thermoneutral conditions of 30°C for 10 days and then either subjected to CE for 2 or 7 days or maintained at 30°C. The mice used in the study by Lelliott et al. (2006) were also born at 22°C, but, unlike the protocol used in our study, they were adapted to thermoneutrality for 3 weeks before the experiment and then exposed to 18°C for 1 week and to 4°C for another 3 weeks (Lelliott et al., 2006).

*Ppargc1a* expression is dramatically elevated already after 6 h of CE (Lin et al., 2002), unlike *Ppargc1b* expression, which is only mildly upregulated after 2-day-CE (see Fig. 3A) or after 3 weeks of CE as described previously (Lelliott et al., 2006). PGC-1α increases the expression of genes for PPARγ and UCP1 (Puigserver et al., 1998), nuclear respiratory factors (NRF1, NRF2), estrogen-related receptors and mitochondrial transcription factor A (mtTFA or TFAM), as well as the nuclear-encoded subunits of the mitochondrial electron transport chain (Liang and Ward, 2006). Thus, the compensatory upregulation of *Ppargc1a* could explain the relatively higher transcript levels for the above genes in PGC-1β-AT-KO mice subjected to CE, possibly reflecting an attempt to compensate for the thermogenic defect by increasing the expression of thermogenesis-related genes (Mottillo et al., 2014), e.g. genes involved in lipid mobilization (*Atgl* and *Plin1*), fatty acid oxidation (*Acadm*, *Acadvl*, *Pdk4* and *Acsl1*), synthesis (*Fasn*) and re-esterification (*Dgat1*), as well mitochondrial biogenesis. Therefore, PGC-1α could upregulate these metabolic pathways in PGC-1β-AT-KO mice (Fig. 3). Because *Ucp1* expression is completely dependent on *Ppargc1a* and not on *Ppargc1b* (Figs 2D and 3), the amount of UCP1 protein required for NST is fully preserved in PGC-1β -AT-KO mice maintained in cold conditions.

The function of PGC-1β is essential, particularly under basal conditions (Lelliott et al., 2006) where its deletion led to increased BAT mass (Table 1). Consistent with lower energy expenditure, and thus lower fuel demands, in the thermoneutral state, in PGC-1β-AT-KO warm-adapted mice relatively high expression of *Cidec* and *Acsl1*, and relatively high levels of PLIN1 were observed, whereas ATGL levels were similar in mice of both genotypes (Fig. 3). CIDEC promotes TAG accumulation into lipid droplets in adipose tissue (Puri et al., 2007), and its interaction with ATGL decreases lipolysis in human adipocytes (Grahn et al., 2014). Overexpression of *Acsl1* in 3T3L1 cells increased fatty acid uptake and incorporation into TAG (Richards et al., 2006), and upregulated *Acsl1* converts free fatty acids into fatty acyl-CoA esters directed toward β-oxidation during CE (Ellis et al., 2010). PLIN1, which is located on the surface of lipid droplets, interacts with CIDEC during TAG accumulation and assists in enlargement of lipid droplets. By this mechanism, PLIN1 counteracts basal lipolysis (Grahn et al., 2013). Thus, upregulation of genes and proteins for these enzymes in BAT of PGC-1β-AT-KO mice likely promotes the occurrence of larger lipid droplets (Fig. 2L,M). This phenotype, induced by adipose tissue-specific *Ppargc1b* ablation in mice, was also observed in brown adipocytes derived from the mutant and WT mice and differentiated *in vitro* in primary culture. Furthermore, under these conditions bigger lipid droplets were detected in adipocytes of the PGC-1β-AT-KO compared with WT genotype, confirming that the morphological changes are dependent on intracellular rather than systemic effects of the gene ablation (data not shown). However, the link between lipid metabolism affected by *Ppargc1b* and BAT mass should be further clarified.

The dynamics of mitochondria, namely their fusion and fission, are closely related to their performance (Mishra and Chan, 2016). PGC-1β is known to regulate *Mfn2* expression through ERRα (encoded by *Esrra*), leading to outer mitochondrial membrane fusion (Liesa et al., 2008). OPA1, a protein associated with the fusion of inner mitochondrial membranes, maintains mitochondrial cristae and is a critical factor connecting mitochondrial morphology and energetics. Both L-OPA1 and S-OPA1 are required for mitochondrial fusion under normal conditions, with L-OPA1 being competent for fusion and S-OPA1 maintaining oxidative phosphorylation function (Lee et al., 2017). However, the functions of the individual OPA-1 forms are still unclear (Lee and Yoon, 2018). OPA1 is localized on lipid droplets in adipocytes and acts as A-kinase anchoring protein, which is responsible for phosphorylation of PLIN1 and induction of lipolysis (Pidoux et al., 2011). Levels of OPA1 and other lipid droplet coating proteins, such as PLIN1, increase during the differentiation of white adipocytes and lipid accumulation (Pidoux et al., 2011). Our observation of lower amounts of the L-form of OPA1 and larger lipid droplets in BAT of PGC-1β-AT-KO mice under thermoneutral conditions indicates impaired communication between mitochondria and lipid droplets, as proven by electron microscopy. The compensatory upregulation of *Ppargc1a* and subsequent activation of lipid and mitochondrial metabolism genes observed in PGC-1β-AT-KO mice exposed to cold conditions (Fig. 3) was apparently insufficient for normal NST induction in BAT (Fig. 2B). As found recently, proteins responsible for mitochondrial dynamics may be involved in adrenergic stimulation of lipolysis. Thus, ablation of mitofusin 2 leads to BAT dysfunction with impaired lipolysis (Boutant et al., 2017; Mahdaviani et al., 2017), reduced respiratory capacity and fat accumulation in BAT. Mitofusin 2 directly interacts with PLIN1 and mediates the functional connection between lipid droplets and mitochondria (Boutant et al., 2017). Our findings suggest a role for PGC-1β in the regulation of lipid metabolism under thermoneutral conditions involving the L-form of OPA1, which could influence adrenergic stimulation of NST in BAT during thermoregulatory thermogenesis.

In summary, our results provide evidence for a unique function of PGC-1β in controlling adrenergically stimulated thermogenesis in BAT by influencing mitochondrial communication with lipid droplets during lipolysis. The role of PGC-1β in regulating the L-form OPA1 and its role in lipolysis deserves further clarification.

## MATERIALS AND METHODS

### Animal design

PGC-1β-AT-KO and their WT mice on C57BL/6 genetic background were generated by crossing mice harboring loxP sites flanking exons 4-5 of the *Ppargc1b* gene (described by Enguix et al., 2013) with mice expressing Cre recombinase under the control of the adiponectin promoter to ensure adipocyte-specific deletion of the gene. The study design has been described previously (Flachs et al., 2017); briefly, 2-month-old-male mice born and raised at 22°C and fed standard chow (extruded ssniff R/M-H from Ssniff Spezialdiaten GmbH, Soest, Germany; metabolizable energy 13 MJ kg^−1^) were acclimatized to 30°C (at thermoneutrality) for 10 days. Thereafter, mice caged in groups of three were randomly divided into three subgroups of similar mean of body weights, maintained on a 12-h light/dark cycle (light from 06:00 h) either at thermoneutrality or exposed to cold (6°C) for 2 or 7 days (Flachs et al., 2017). The mice had free access to food and water throughout the duration of the experiment. Food consumption and body weight were monitored daily. At the end of experiments, mice were killed in random-fed state by cervical dislocation (between 09:00 h and 10:00 h) under ether anesthesia. EDTA-plasma and tissues were collected, including iBAT, eWAT, scWAT, liver and quadriceps skeletal muscle. Samples were flash-frozen and stored in liquid nitrogen for other analysis. The sample size was set to *n*=5-8 based on previous experiences and all the experiments were at least twice repeated. All experiments were approved by the Animal Care and Use Committee of the Institute of Physiology of the Czech Academy of Sciences (Approval Number 81/2016) and followed the guidelines for the use and care of laboratory animals of the Institute of Physiology.

### Plasma parameters and tissue lipid content

Plasma levels of insulin, glucose, NEFA and total TAG were evaluated as described (Flachs et al., 2017). TAG content in liver and quadriceps skeletal muscle was determined in ethanolic KOH tissue lysates (Flachs et al., 2017). TAG content was estimated using gravimetry in BAT extracts (Bardova et al., 2020).

### Indirect calorimetry

Both cold tolerance and the NE-test were performed using an indirect calorimetry system (INCA; Somedic, Horby, Sweden) (Kus et al., 2008). Five-week-old mice were used for implantation of E-mitters for detection of core body temperature during the cold tolerance test. After 1 week of recovery at 22°C, mice were exposed to a thermoneutral temperature of 30°C for at least for 10 days. Thereafter, the core body temperature was measured using INCA at an ambient temperature of 6°C. Any mouse showing hypothermia (core body temperature <28°C) was rescued from the cold conditions. During the test, no food or water were provided.

The NE test was performed on pentobarbital-anaesthetized (100 mg per kg of body weight) mice exposed to a thermoneutral temperature of 30°C or cold conditions (6°C; 7-day-CE) as described above. The maximal stimulation of whole-body oxygen consumption in response to NE (1 mg per kg of body weight) or saline solution (0.9% NaCl) used as a control (Fig. S4) was measured at 33°C using INCA system (Golozoubova et al., 2006). Data were evaluated as a delta area under the curve (ΔAUC) of the oxygen consumption curves calculated as the difference between the AUC after intraperitoneal NE injection and the AUC at baseline (30 min before NE application).

### RNA isolation and gene expression analysis

Total RNA was isolated from iBAT samples using Tri Reagent (Sigma-Aldrich) and transcript levels were evaluated by RT-qPCR using a LightCycler480 (Roche) as described (Flachs et al., 2017). Data were normalized to the geometrical mean of three reference genes (18 s ribosomal RNA, *Rn18s*; β-2 microglobulin, *B2m*; and hypoxantine guanine phosphoribosyl transferase, *Hprt*). For PCR primer sequences, see Table S2.

### Immunohistochemical analysis of BAT and scWAT

Formalin-fixed paraffin-embedded sections (3 µm) were prepared as described previously (Flachs et al., 2017). The size of BAT adipocytes was assessed using an antibody against sodium potassium ATPase (1:1000; ab76020, Abcam), which stains the plasma membrane, and the size of lipid droplets was detected using anti-perilipin 1 antibody (1:200; ab61682, Abcam), which stains the surface of lipid droplets. Only lipid droplets larger than 3 µm^2^ were evaluated (magnificence limitation). The morphometry data are based on measurements of 1000 brown adipocytes and 800 lipid droplets taken randomly from ten different sections per animal. UCP1 immunohistochemical staining was performed as described previously (Teodoro et al., 2014). Twelve images were taken in comparable areas on whole-tissue sections of scWAT (dorsolumbal WAT). Ratios of UCP1-positive and -negative areas were calculated. Digital images were captured using an Olympus AX light microscope and a Leica TCS SP8 confocal microscope. All analyses were performed using the imaging software NIS-Elements AR3.0 (Laboratory Imaging, Prague, Czech Republic).

### Protein extraction and western blotting analysis

BAT tissue homogenate was prepared from frozen samples using a glass-Teflon homogenizer in STE medium (250 mM sucrose; 50 mM Tris; 5 mM Na_2_-EDTA; 1 mg/ml aprotonin; 1 mg/ml pepstatin; 1 mg/ml leupeptin; 1 mM phenylmethylsulfonylfluoride; pH 7.5) and centrifugation (600 ***g***, 4°C, 10 min). The protein concentration in the tissue homogenate was determined using a bicinchoninic acid assay. The content of UCP1 (1:1000; R&D Systems, MAB 6158), OPA1 (1:2000; BD Biosciences, 612606), mitofusin 2 (1:1000; Abcam, ab56889), DRP1 (1:500; Santa Cruz Biotechnology, sc-32898), PLIN1 (1:1000; Abcam, ab1682) and ATGL (1:1000; Cell Signaling Technology, #2138S) was analyzed by western blotting as described (Flachs et al., 2017; Pecinova et al., 2011). Infra-red dye-labeled antibodies were used as secondary antibodies and signals were quantified using the Odyssey IR Imaging System (Li-Cor Biosciences). Signals on different blots were compared using a standard [St1 (half concentration of St2) and St2] prepared from BAT homogenate of C57BL/6 mice exposed to 6°C for 7 days. Signal analyses were performed using ImageLab software (Bio-Rad Laboratories). For quantification, the original sample signal was divided by the sum of the signals of the two standards (St1 and St2) and multiplied by the ratio of the signals of the standards. Data are expressed in arbitrary units. For loading controls, the signal of antibodies specific for α-tubulin (1:1000; Cell Signaling Technology, 2125S), glyceraldehyde-3-phosphate dehydrogenase (GAPDH; 1:1000; Cell Signaling Technology, 2118S) or citrate synthase (1:1000; Abcam, Ab129095) were used (see Figs 2E, 3C and 4A).

### Electron microscopy

Samples of brown adipose tissue were fixed with 2% glutaraldehyde and 2% formaldehyde in 0.1 M Na/K phosphate buffer, pH 7.2-7.4 (Sörensen's buffer, SB). After fixation for 1.5 h at 4°C, the tissue was quickly cut into very small pieces and put back into the same fixative overnight at 4°C. Samples were then incubated with 0.02 M glycine in SB for 30 min and postfixed with 1% OsO_4_ in SB for 1.5 h in the dark at 4°C.

Afterwards, samples were dehydrated in series of ethanol, further dehydrated with propylene oxide and embedded in Quetol-NSA resin. After polymerization for 72 h at 60°C, resin blocks with were cut into 85 nm ultrathin sections and collected on 200 mesh size copper grids. Sections were examined in a JEOL JEM-1400Flash transmission electron microscope operated at 80 kV equipped with Matataki Flash sCMOS camera (JEOL Ltd.). For each mouse, ten resin blocks with embedded tissue were processed, of which up to three were examined (for details, see Cinti, 2001).

The program NIS Elements AR3.0 was used for evaluation of images. Twenty images per sample at 3000× magnification were used to assess the length of both mitochondria and contacts between mitochondria and lipid droplets, and at 1200× magnification for evaluation of lipid droplet size. The mean length of mitochondria was calculated from the length of ten randomly chosen mitochondria per image. Contact between mitochondria and lipid droplets was expressed as a percentage of the contact length between mitochondria to the total surface of lipid droplets per image. The size of lipid droplets was evaluated only in samples from cold-adapted mice (because of the magnification of pictures and large size of lipid droplets at thermoneutrality) and the size of ten lipid droplets per image was measured.

### Statistical analyses

Data were analyzed by analysis of variance (*t*-test, one-way or two-way ANOVA). SigmaStat 3.5 software (Systat Software) was used for statistical evaluation. Logarithmic or square root transformations were used to stabilize variance or normality of samples when necessary and to ensure that data met the assumption of the tests (equal variance and normal distribution). All values are presented as mean±s.e.m. and comparisons were judged to be significant at *P*<0.05. Data were visualized using GraphPad Prism (Version 8.3.1, 2019).

## Supplementary Material

Supplementary information
