# Supplementary material for: Adipose tissue-specific ablation of PGC-1β impairs thermogenesis in brown fat
Source: Dis Model Mech. 2022 Apr 25;15(4):dmm049223. doi: 10.1242/dmm.049223 (PMC9066513; doi:10.1242/dmm.049223)
Supplement: Supplementary information [file dmm-15-049223-s1.pdf]

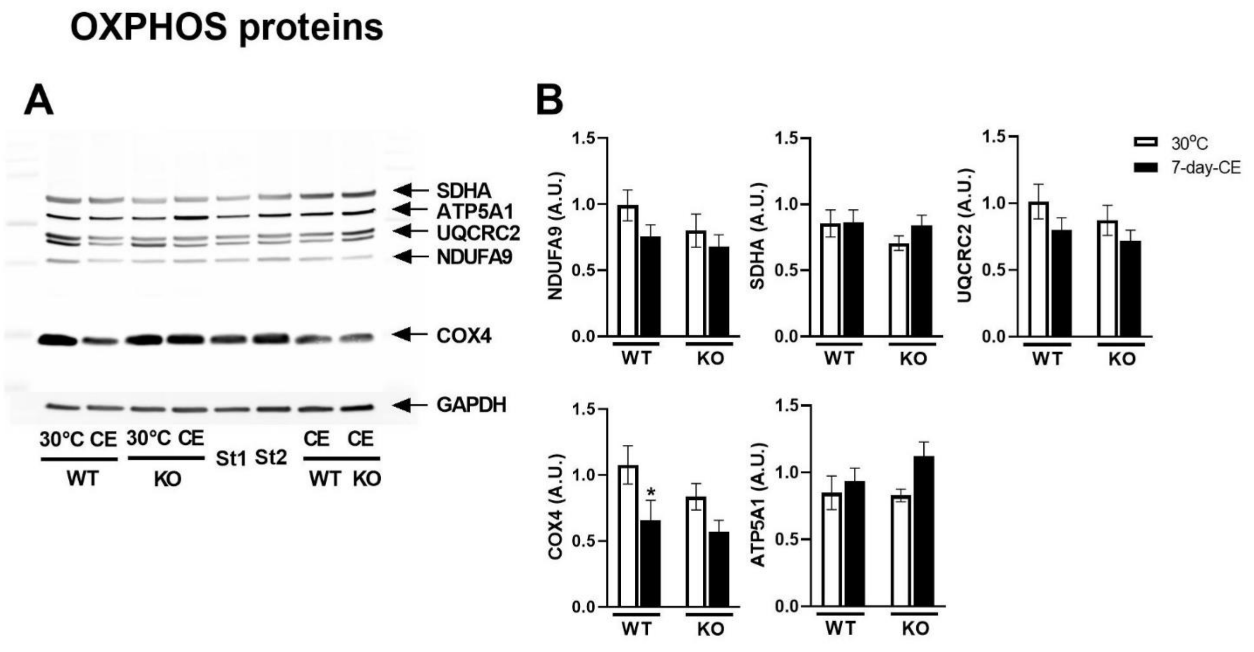

Fig. S1. OXPHOS proteins in interscapular BAT. (A) Representative blots of OXPHOS proteins (NDUFA9, complex I; SDHA, complex II; UQCRC2, complex III; COX4, complex IV; ATP5A1, complex V) and glyceraldehyde-3-phosphate dehydrogenase (GAPDH; used as a loading control). Standards St1 and St2 (St1 is a half concentration of St2) were used for comparison of signals on different blots. (B) Amount of OXPHOS proteins per 10 μg of BAT homogenate proteins. Data are expressed in arbitrary units (A.U.). n=9. Data are means±s.e.m. Statistical analysis was performed using two-way ANOVA ($P<0.05$) *Significant difference compared to 30°C within the genotype. KO, PGC-1β-AT-KO mice; WT, wild-type mice, 7-day-CE, mice exposed to cold for 7 days; 30°C, mice adapted to thermoneutral conditions.

Disease Models & Mechanisms • Supplementary information

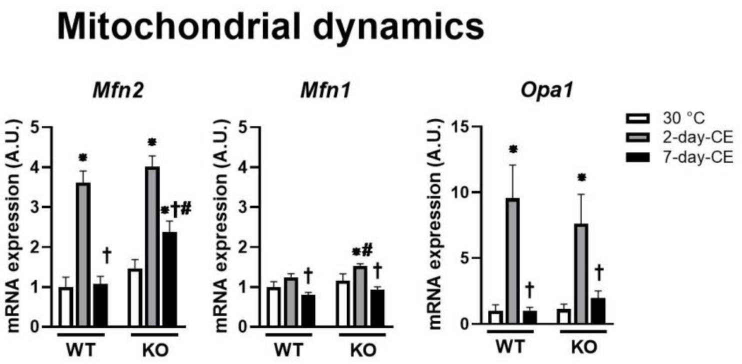

Fig. S2. Effect of PGC-1β deletion on the expression of genes involved in mitochondrial dynamics. Data are expressed in arbitrary units (A.U.). n=8. Data are means±s.e.m. Statistical analysis was performed using two-way ANOVA (P<0.05) * Significant differences compared to 30°C within the genotype; □ significant differences compared to 2 days at 6°C within the genotype; #significant differences between genotypes within the same temperature. KO, PGC-1β-AT-KO mice; WT, wild-type mice; 30°C, mice adapted to thermoneutral conditions; 2-day-CE, mice exposed to cold for 2 days; 7-day-CE, mice exposed to cold for 7 days; 30°C, mice adapted to thermoneutral conditions.

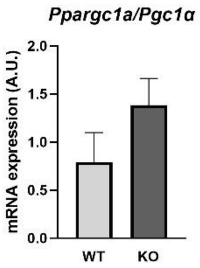

Fig. S3. Effect of PGC-1β deletion on the expression of *Ppargc1a* gene in BAT of mice maintained at 22° C. Data are expressed in arbitrary units (A.U.). n=9. Data are means±s.e.m. KO, PGC-1β-AT-KO mice; WT, wild-type mice.

Disease Models & Mechanisms • Supplementary information

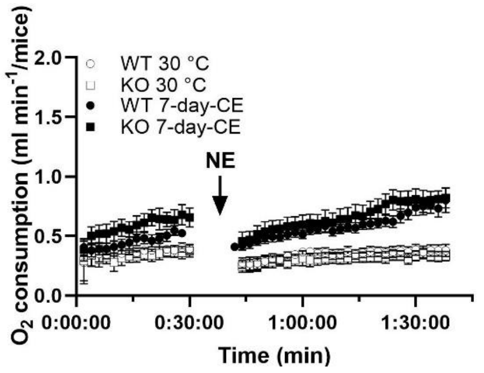

Fig. S4. Whole-body oxygen consumption during NE test in control mice. Oxygen consumption in control mice was measured at 33°C using INCA system in response to saline solution (0.9% NaCl). n=5. Data are means±s.e.m. KO, PGC-1β-AT-KO mice; WT, wild-type mice; 7-day-CE, mice exposed to cold for 7 days; 30°C, mice adapted to thermoneutral conditions; NE, norepinephrine

Disease Models & Mechanisms • Supplementary information

Table S1. General characterization of PGC-1β-AT-KO and WT mice fed standard chow, high-fat diet or calorie restricted high-fat diet and maintained at 22 °C.

	WT			*KO*		
	STD	*cHF*	*cHF-R*	*STD*	*cHF*	*cHF-R*
Body weight (g)	27.72 ± 0.97	40.84 ± 2.09[a]	34.30 ± 1.4[ab]	29.27 ± 0.60	43.99 ± 2.53[a]	36.72 ± 0.93[ab]
Body weight gain (g)	0.98 ± 0.52	11.75 ± 1.42[a]	5.39 ± 0.75[ab]	1.38 ± 0.26	14.01 ± 1.6[a]	7.27 ± 0.7[ab]
Weight of AT depots						
eWAT (mg)	318 ± 45	2265 ± 244[a]	1655 ± 242[ab]	410 ± 43	2564 ± 281[a]	1824 ± 129[ab]
scWAT (mg)	194 ± 21	1105 ± 139[a]	739 ± 109[ab]	262 ± 20	1402 ± 165[ac]	911 ± 67[ab]
BAT (mg)	88 ± 6	165 ± 14[a]	120 ± 11[ab]	118 ± 5[c]	224 ± 24[ac]	169 ± 17[abc]
Plasma levels						
TAG (mmol l^{-1})	84 ± 7	93 ± 11	58 ± 4[ab]	79 ± 7	102 ± 10	69 ± 9[b]
NEFA (mmol l^{-1})	0.53 ± 0.03	0.56 ± 0.04	0.47 ± 0.02	0.50 ± 0.04	0.53 ± 0.03	0.47 ± 0.02
Glucose (mg dl^{-1})	7.3 ± 0.3	8.7 0.3[a]	8.7 ± 0.3[a]	7.9 ± 0.4	8.8 ± 0.4	8.9 ± 0.4

Two-month-old-male mice born and raised at 22 °C and fed standard chow (extruded ssniff R/M-H from Ssniff Spezialdiaten GmbH, Soest, Germany; metabolizable energy 13 MJ kg^{-1}) were fed either the standard (STD) or high fat diet based on corn oil (cHF; lipid content, 35% wt/wt, mainly corn oil, (Kuda et al., 2009)) for another 6 weeks. Some cHF diet-fed mice were subjected to 10% calorie restriction (cHF-R), as described before (Flachs et al., 2011). Mice were killed in random-fed state by cervical dislocation (between 9 – 10 a.m.) under ether anesthesia and dissected as described in the main text.

Abbreviations: AT, adipose tissue; eWAT, epididymal white adipose tissue; BAT, interscapular brown adipose tissue; NEFA, non-esterified fatty acid; scWAT, subcutaneous white adipose tissue; TAG, triacylglycerol. Mice were fed chow (STD) or high-fat diet (cHF) or cHF with 10% caloric restriction. Data are means ± SEM; n = 9. [a]Significant differences as compared with STD within the genotype. [b]Significant differences as compared with cHF within the genotype. [c]Significant differences between genotypes within the treatment (ANOVA).

Table S2. Primer sequencing of genes detected using qPCR.

Gene name	Gene ID	Forward primer	Reverse primer
Acadm	11364	TCGCCCCGGAATATGACAAAA	GCCAAGGCCACCGCAACT
Acadvl	11370	CAGGGGTGGAGCGTGTGC	CATTGCCCAGCCCAGTGAGTTCC
Aco2	11429	TCTCTAACAACCTGCTCATCGG	TCATCTCCAATCACCACCCACC
Acsl1	14081	GAAGCCGTGGCCCAGGTGTTTGTC	TTCGCCTTCAGTGTTGGAGTCAGA
Atp5b	11947	TTCACGGGTCACATGGGGAAGC	TCACGACCCGTGCTCTTCTGC
Atp5o	28080	AAGGACCCCAAAGTGTCTCTG	TAGGCGACCATTTTCAGCAAG
B2m	12010	AATTGAAGTCTGTCACTGTGCCCAA	ACAAAAGCAGAAGTAGCCACAGGGT
Cidec	14311	GACAAGCCCTTCTCCCTGGTG	TCTCTTCTTGCGCTGTTCTGATGG
Cox4i1	12857	TTGGCTTCACTGCGCTCGTTC	GGCCACCCAGTCACGATCGAA
Cox5a	12858	AGGTTGTTAAGGACAAAGCAGGACC	TCAAGGCCCAGCTCCTCTGGA
Cox5b	12859	GTGGGCTGCATCTGTGAAGAGGA	TCCACAGTTGGGGCATCGCT
Cox7a1	12865	GACAATGACCTCCCAGTACAC	GCCCAGCCCAAGCAGTATAAG
Cyc1	66445	ATTTCAACCCTTACTTTCCCG	CCACTTATGCCGCTTCATGGC
Dgat1	13350	TGGCCAGGACAGGAGTATTTTTGA	CTCGGGCATCGTAGTTGAGCA
Esrra	26379	CAGGATCTGCCCAGCATAGG	GCTTCTGACAATCCCCCACA
Fasn	14104	TGGGTGTGGAAGTTCGTCAG	GTCGTGTCAGTAGCCGAGTC
Hprt	15452	GCTGAGGCGGCGAGGGAGAG	GCTAATCACGACGCTGGGACTGC
Mfn1	67414	TGGGTGCTGGGTTGCAGTATTCA	CGGGAGCAAACCCAGAGAACCA
Mfn2	170731	GGTCAAAGCAGGGAGGATGCCA	AGACTGAGGGGCGAGTGAGCA
Mdh2	17448	TGGACGCCATGAACGGGAAGG	TTTCCCCAAGAGCAAGGGCGT
Ndufab1	70316	GGAATCAAGGACCGAGTTCTG	TCCAAACTGTCTAAGCCCAGG
Nrf1	18181	TGGCGCAGCACCTTTGGAGA	CCCCCGACCTGTGGAATACTTGA
Opa1	74143	AGACTGTGTCAAAATCCTGCTCA	GCTGCAGGTTAAGTGCCTGGA
Pdk4	27273	GGCTTGCCAATTTCTCGTCTCTA	TTCGCCAGGTTCTTCGGTTCC
Plin1	103968	CTATGTGCCGCTTCCCAGGCT	CGCCTCGGTTTTGTCGTCCAG
Pnpla2	66853	GGCAATCAGCAGGCAGGGTCTTTA	GCCAACGCCACTCACATCTACG
Ppara	19013	TGCGCAGCTCGTACAGGTCATCAA	CCCCCATTTCGGTAGCAGGTAGTCTTA
Pparg	19016	GCCTTGCTGTGGGGATGTCTC	CTCGCCTTGGCTTTGGTCAG
Ppargc1a	19017	CCCAAAGGATGCGCTCTCGTT	TGCGGTGTCTGTAGTGGCTTGATT
Ppargc1b	170826	AGTGGGTGCGGAGACACAGATG	GTATGGAGGTGTGGTGGGTGGC
Rna18s	19791	GCCCGAGCCGCCTGGATAC	CCGGCGGGTCATGGGAATAAC
Sdhb	67680	GACCTGCCCCAAGGGTCTGA	TTGACAGGCACACTCAGCACGG
Tfam	21780	AGGAGCTGAAGGCATGCGGTGAAG	GTCCAGTGCGTGCGGGTGAAC
Ucp1	22227	CACGGGGACCTACAATGCTTACAG	GGCCGTCGGTCCTTCCTT
Uqcrh	66576	TTGCATGCACGGGACCACTGT	ACCGACGTCCAGAACCACAAGG

Gene names abbreviations: *Acadm*, acyl-Coenzyme A dehydrogenase, medium chain; *Acadvl*, acyl-Coenzyme A dehydrogenase, very long chain; *Aco2*, aconitase 2; *Acsl1*, acyl-CoA synthetase long-chain family member 1; *Atp5b*, ATP Synthase F1 subunit beta; *Atp5o*, ATP synthase subunit O (both ATPase); *B2M*, β-2 microglobulin; *Cidec*, cell death inducing DFFA like effector C; *Cox4i1*,

cytochrome C oxidase subunit 4I1; cytochrome C1; *Cox5a*, cytochrome C oxidase subunit 5A; *Cox5b*, cytochrome C oxidase subunit 5B; *Cox7a1*, cytochrome C oxidase subunit 7A1; *Cyc1*,; (all Complex IV); *Dgat1*, diacylglycerol O-acyltransferase 1; *Esrra*, estrogen related receptor alpha; *Fasn/Fas*, fatty acid synthase; *Hprt*, hypoxantine guanine phosphorybosil transferase; *Mfn1*, mitofusin 1; *Mfn2*, mitofusin 2; *Mdh2*, malate dehydrogenase 2; *Ndufab1*, NADH:ubiquinone oxidoreductase subunit AB1; *Nrf1*, nuclear respiratory factor 1; *Opa1*, Optic Atrophy Protein 1; *Pdk4*, pyruvate dehydrogenase kinase, isoenzyme 4; *Pnpla2/Atgl*, adipose triglyceride lipase, also known as patatin-like phospholipase domain-containing protein 2*Plin1*, perilipin 1; *Ppara*, peroxisome proliferator activated receptor alpha; *Pparg*, peroxisome proliferator activated receptor gamma; *Ppargc1b*, peroxisome proliferative activated receptor gamma, coactivator 1 beta; *Ppargc1a*, peroxisome proliferative activated receptor gamma, coactivator 1 alpha; *Rna18s*,18 s ribosomal RNA; *Sdhb*, succinate dehydrogenase complex; *Tfam*, transcription factor A, mitochondrial;); Ucp1, uncoupling protein; *Uqcrh*, ubiquinol-cytochrome C reductase.

Flachs, P., Ruhl, R., Hensler, M., Janovska, P., Zouhar, P., Kus, V., Macek Jilkova, Z., Papp, E., Kuda, O., Svobodova, M. et al. (2011). Synergistic induction of lipid catabolism and anti-inflammatory lipids in white fat of dietary obese mice in response to calorie restriction and n-3 fatty acids. *Diabetologia* **54**, 2626-38.

Kuda, O., Jelenik, T., Jilkova, Z., Flachs, P., Rossmeisl, M., Hensler, M., Kazdova, L., Ogston, N., Baranowski, M., Gorski, J. et al. (2009). n-3 fatty acids and rosiglitazone improve insulin sensitivity through additive stimulatory effects on muscle glycogen synthesis in mice fed a high-fat diet. *Diabetologia* **52**, 941-51.

Disease Models & Mechanisms • Supplementary information
